# Intrinsic and Extrinsic Modulators of the Epithelial to Mesenchymal Transition: Driving the Fate of Tumor Microenvironment

**DOI:** 10.3389/fonc.2020.01122

**Published:** 2020-07-24

**Authors:** Edoardo D'Angelo, Rafael Soares Lindoso, Francesca Sensi, Salvatore Pucciarelli, Benedetta Bussolati, Marco Agostini, Federica Collino

**Affiliations:** ^1^First Surgical Clinic, Department of Surgery, Oncology and Gastroenterology, University of Padova, Padua, Italy; ^2^LIFELAB Program, Consorzio per la Ricerca Sanitaria–CORIS, Veneto Region, Padua, Italy; ^3^Institute of Pediatric Research, Fondazione Citta della Speranza, Padua, Italy; ^4^Institute of Biophysics Carlos Chagas Filho, Federal University of Rio de Janeiro, Rio de Janeiro, Brazil; ^5^National Institute of Science and Technology for Regenerative Medicine–REGENERA, Federal University of Rio de Janeiro, Rio de Janeiro, Brazil; ^6^Division of Pharmacology, Utrecht Institute for Pharmaceutical Sciences, Utrecht University, Utrecht, Netherlands; ^7^Department of Molecular Sciences and Nanosystems, Cà Foscari University of Venice, Venice, Italy; ^8^Department of Medical Sciences, Molecular Biotechnology Center, University of Torino, Turin, Italy; ^9^Department of Biomedical Sciences, University of Padova, Padua, Italy; ^10^Pediatric Nephrology, Dialysis and Transplant Unit, Fondazione Ca' Granda, IRCCS Policlinico di Milano, Milan, Italy

**Keywords:** epithelial to mesenchymal transition, tumor microenvironment, extracellular matrix, extracellular vesicles, personalized medicine

## Abstract

The epithelial to mesenchymal transition (EMT) is an evolutionarily conserved process. In cancer, EMT can activate biochemical changes in tumor cells that enable the destruction of the cellular polarity, leading to the acquisition of invasive capabilities. EMT regulation can be triggered by intrinsic and extrinsic signaling, allowing the tumor to adapt to the microenvironment demand in the different stages of tumor progression. In concomitance, tumor cells undergoing EMT actively interact with the surrounding tumor microenvironment (TME) constituted by cell components and extracellular matrix as well as cell secretome elements. As a result, the TME is in turn modulated by the EMT process toward an aggressive behavior. The current review presents the intrinsic and extrinsic modulators of EMT and their relationship with the TME, focusing on the non-cell-derived components, such as secreted metabolites, extracellular matrix, as well as extracellular vesicles. Moreover, we explore how these modulators can be suitable targets for anticancer therapy and personalized medicine.

## Epithelial to Mesenchymal Transition in Cancer Progression

Epithelial to mesenchymal transition (EMT) has been described as an evolutionarily conserved process, where polarized epithelial cells undergo phenotypical changes and assume a motile migrating mesenchymal cell phenotype (1). Such process is involved in different contexts in the organisms and therefore is classified in three types based on the regulatory molecules involved, the microenvironment and the EMT role in a specific tissue: (i) EMT type I is crucial during embryonic development and in the formation of tissues and organs, generating primary mesenchyme that posteriorly can undergo mesenchymal to epithelial transition and lead to the formation of secondary epithelia (2). (ii) EMT type II is involved with wound healing, tissue regeneration, and the fibrotic process. Different from type I, EMT type II is responsive to the inflammatory process and therefore is associated with chronic diseases that, in long terms, can result in the impairment of organ functions and its failure (3, 4). (iii) EMT type III characterizes a biological process that occurs in neoplastic cells and represents a key element in tumor migration and metastasis development. Different from embryogenesis, the mechanism associated with the regulation of such a process presents distinct molecular signatures where genetically abnormal cells do not respond to normal growth regulatory signals (5, 6).

**Figure 1:**
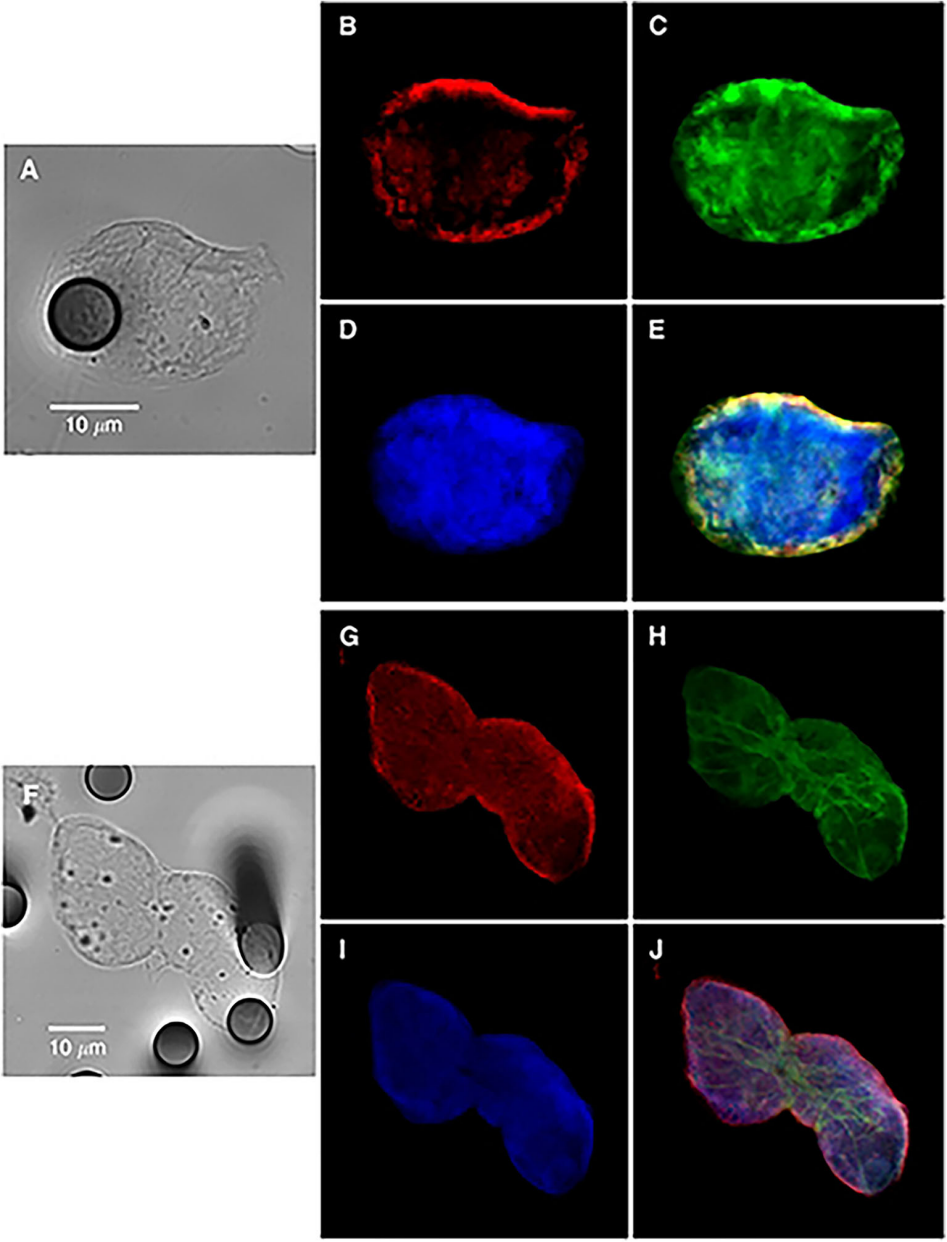
Bright-field and immunofluorescence analysis of circulating tumor cells from a non-small-cell lung cancer patient (single cell in **A–E** or cluster in **F–J)**, showing hybrid-phenotype cells expressing both epithelial and mesenchymal markers. Keratins (**B,G**, red); vimentin (**C,H**, green), and nucleus **(D,I)**. **(E,J)** images are merged panels. Contribution from Lecharpentier et al. (10).

During the EMT process in cancer, epithelial cells lose their polarity and the cell–cell interactions (like adhesion molecules—E-cadherin and cytokeratins—tight junctions and gap junctions) to acquire a more mesenchymal phenotype, presenting increased migratory and invasiveness capacity, apoptosis resistance, and enhanced extracellular matrix (ECM) secretion (7, 8). Moreover, EMT has been shown to increase the activity of matrix metalloproteinases (MMPs), leading to ECM remodeling which corroborates to cell motility and invasion (9). It is important to take into consideration the fact that EMT is not a complete process in cancer resulting in a cell with a hybrid phenotype that expresses epithelial and mesenchymal genes (10) (Figure 1), leading to a more aggressive behavior that facilitates the development of secondary tumors (11). The wide extension of EMT modifications also provides to the tumor the acquisition of drug resistance (12). Although the mechanisms were not entirely elucidated, EMT seems to trigger different pathways that promote the increase of drug efflux pumps and anti-apoptotic effects (13). Such properties have been of great interest as a possible therapeutic target against cancer (14). In addition, the cancer stem cells (CSCs) concept has been connected to the EMT process, supporting the notion of stemness as a temporary flexible characteristic of tumor cells that can be lost and regained (15, 16). Cancer cells that undergo EMT gain stem cell-related capabilities and mesenchymal traits together with enhanced capacity to generate spheroids *in vitro* and to be tumorigenic *in vivo* (15).

The regulation of EMT is a complex process and can be triggered by different components present in the tumor microenvironment (TME) like inflammation, hypoxia, and secreted bioactive molecules (17). In particular, EMT-dependent invasion and metastatic programs in tumor cells are strongly influenced by the TME, which can facilitate cell extravasation from the primary tumor and cancer therapy resistance (18). Moreover, in the past years, the metastatic process has been reconsidered as a heterogeneous and adaptive activity (19), in which tumor cells and the stroma influence one another in a reciprocal manner, mutually supporting cancer progression (19).

In this review, we summarize the more relevant intrinsic and extrinsic signals affecting metabolic reprogramming and EMT process in cancer cells. Moreover, we dissect the complex interaction between tumor cells and the surrounding TME components and how they can be modulated by the EMT process toward tumor progression and metastasis.

## Signals Promoting Epithelial to Mesenchymal Transition

### Intrinsic Signals—Metabolic Pathways and Epithelial to Mesenchymal Transition

During primary and then metastatic neoplastic transformation, tumor cells have to adapt their metabolism according to environmental changes (20). Recently, many studies have highlighted how the reprogramming of cancer cell metabolism and the processes of EMT are closely interconnected (21).

Cancer cell metabolism is characterized by improved utilization of glucose, a phenomenon known as the Warburg effect, a characteristic metabolic alteration of cancer cells (22–24). Glucose transporter (GLUT)1 is induced by hypoxia-inducible factor 1α (HIF-1α) increase during cancer progression (25, 26). Overexpression of GLUT1 increases MMP-2 expression both *in vitro* and *in vivo*, which is essential for EMT (27–29). Differently, GLUT3 is a transcriptional target of the Zinc finger E-box-binding homeobox 1 (Zeb1), an EMT marker. GLUT3 expression in lung cancer patients has been shown to correlate with poor patient survival (30). Hexokinase 2 (HK2), a well-known hypoxia-inducible gene, has been described to be upregulated in different types of brain cancers and correlated with EMT (31, 32). In particular, its upregulation increases the expression of the EMT marker Snail Family Transcriptional Repressor 1 (Snai1) (33). Another enzyme is aldolase A (ALDOA), linked with the stimulation of mesenchymal markers in lung carcinoma (34). Furthermore, glyceraldehyde-3-phosphate dehydrogenase (GAPDH) shows a crucial role in metabolism and gene transcription. In colon cancer, the silencing of GAPDH expression resulted in a reduction of Snai1, leading to inhibition of EMT and attenuation of cell migration (35).

Dysfunctions in mitochondria, in particular, downregulation of mitochondrial genes, is a common feature of highly aggressive cancers and significantly correlates with the activation of EMT signals (36, 37). Some enzymes involved in the tricarboxylic acid cycle (TCA) are linked with EMT. For example, mutations in succinate dehydrogenase (SDH), a component of the respiratory chain that transforms succinate to fumarate, have been described in pheochromocytomas, paragangliomas (38–40), and gastrointestinal stromal neoplasia (41, 42). A recent study by Guo (43) revealed that transforming growth factor-beta 1 (TGFβ1) treatment can induce mitochondrial morphologic variations in connection with a shift from epithelial to mesenchymal phenotype of pancreatic cancer cells.

**Figure 2:**
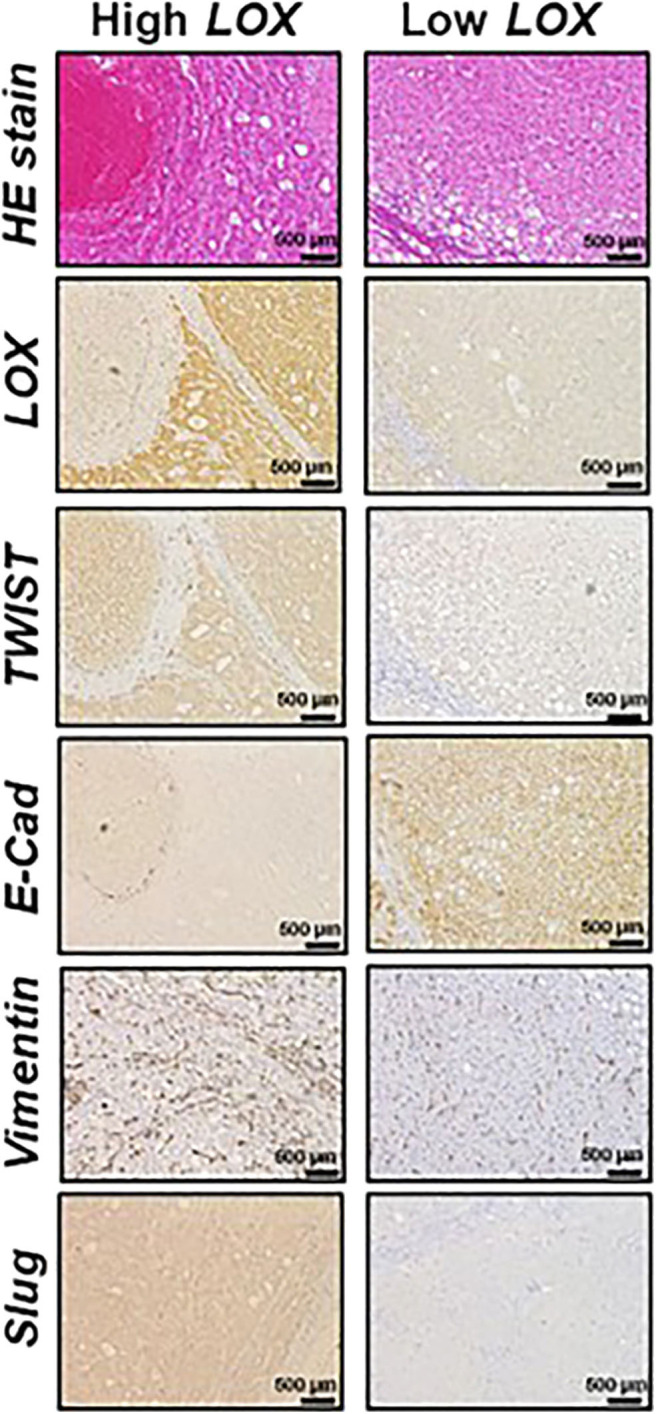
Representative histological analysis [hematoxilin and eosin (HE)] and immunohistochemistry of lysyl oxidase (LOX), TWIST, E-cadherin (E-Cad), vimentin and Slug in hepatocellular carcinoma (HCC) expressing high (left panels) and low (right panels) LOX levels. Contribution from Umezaki et al. (49).

Furthermore, dysfunctions in lipid metabolism are also connected with EMT. For instance, it has been demonstrated that the overexpression of acetyl-CoA synthetase (ACSL1 and ACSL4) and stearoyl-CoA desaturase (SCD) activates EMT, increasing migration, invasion, and colony formation (44). From the clinical point of view, the high expression of ACSL1, ACSL4, and SCD is associated with poor prognosis in colorectal cancer patients (45). Moreover, it has been observed that ATP citrate lyase (ACL) reverses EMT in non-small-cell lung cancer cell lines by Snai1 repression (46). Furthermore, the role of lysyl oxidase (LOX) family in EMT promotion is also important. LOX expression is regulated by HIF factors and often upregulated in metastatic tumors (47, 48). Patients with a high expression of LOX in tumors have poor overall survival (Figure 2) (49, 50). Conversely, 5-LOX which catalyzed the conversion of arachidonic acid to lipoxin A4, functions as an EMT suppressor (51), supporting alternative antitumor activity of LOX substrates.

The amino acid metabolism also plays an important role in maintaining cellular metabolic homeostasis. Altered amino acid metabolism is also involved in the mechanisms of EMT regulation. Glutamine is known as the most abundant amino acid with nutrient functions involved in multiple phases of cancer metabolism (52). The relation between glutamine and EMT activation has recently been demonstrated in a study in which inhibition of glutaminase 1 pathway reduces lung metastasis formation by the repression of Snai1 (53). Moreover, asparagine bioavailability intensely influences tumor potential (54). The knockdown of asparagine in an *in vivo* breast cancer model induces the alteration of Twist Family BHLH Transcription Factor 1 (Twist1) and E-cadherin expression only at the metastasis site, which indicates an impaired EMT behavior (54). Indoleamine 2,3-dioxygenase 1 (IDO1) is a central enzyme in tryptophan metabolism. High levels of IDO1 have been found in different human tumor tissues as lung (55), colorectal (56, 57), and bladder (58) cancers, where its reduction has been correlated to EMT inhibition (58).

One of the well-known crucial pathways in tumor dissemination is the Hippo signaling pathway. Glycolis, the most used ATP supplier system in invasive cancer cells, has been described to strongly regulate the Hippo-downstream interacting proteins, YES-associated protein (YAP), and its partner, the transcriptional coactivator with PDZ-binding motif (TAZ) (59, 60). Wang et al. (60) demonstrated that glucose deprivation in cancer cells can activate large tumor suppressor kinase (LATS) and AMP-activated protein kinase (AMPK), which in turn phosphorylate YAP, contributing to its inactivation. On the other hand, YAP stimulated GLUT3 expression at the transcriptional level, inducing glucose metabolism and lactate production in cancer cells (60). The YAP/TAZ pathway is also involved in amino acid-dependent activation of mammalian target of rapamycin complex (mTORC)1, mediating tumor biosynthesis and growth (61). In particular, YAP/TAZ knockout cells were unable to activate the high-affinity amino acid transporter LAT1, blocking leucine uptake and cancer cell aggressive growth advantage (61). Lastly, Sorrentino et al. (62) reported a role of sterol regulatory element-binding protein (SREBP)/mevalonate pathway in the activation of YAP/TAZ pathway both in MDA-MB-231 and MCF10A breast cancer cell lines, impacting tumor proliferation and self-renewal properties.

Downregulation of Hippo pathway components has been observed in various human cancers and strongly correlated with EMT and aggressiveness (63). Morvaridi et al. (64) demonstrated that activated pancreatic stellate cells show an increased expression of YAP and TAZ proteins and actively participate in the metastatic process. In addition, Yuan et al. (65) proposed the YAP/TAZ-dependent AKT upregulation in pancreatic cancer, one of the principal mechanisms involved in the resistance of gemcitabine treatment. There is a broad and rapidly growing literature which shows how dysregulated Hippo pathway extensively affects the TGFβ, Wnt, Sonic hedgehog, and Notch signaling, which are not the focus of this review, but are reviewed in depth elsewhere (66, 67).

## Tumor Microenvironment-Derived Extrinsic Signals Promoting Epithelial to Mesenchymal Transition

### Stromal Cells

Today, it is well-known that TME consists of different stromal players, which coevolve with cancer cells and contribute to cancer progression and metastasis: fibroblast (68), immune cells (69), and endothelial cells (70). These “accessories to the crime” stromal cells (71) are recruited by cancer cells through stimulatory growth factor, cytokines, and chemokine. In turn, attracted stromal cells foster cancer cell progression by secreting growth factor, ECM-remodeling, enzymes and essential intermediate metabolites (71).

The most involved stromal cells in cancer progression and EMT stimulation are represented by fibroblasts. Fibroblasts during healthy homeostatic processes are critical for epithelial steady-state maintenance and essential during wound healing. During his histological studies, Dvorak (72), describing the tumor as “wounds which do not heal,” seminally suggested a possible involvement of fibroblasts in carcinogenesis. In the TME, the main source of cancer-associated fibroblasts (CAFs) derives from normal fibroblasts accumulating directly genetic and epigenetic alterations. However, recently, CAFs have been demonstrated to be generated from endothelial cells, epithelial cells, and cancer cells (73). For example, CAFs may originate from fibroblasts exposed to chronical oxidative stress (74) or from senescent cells. Wang et al. (75) demonstrated that CAFs isolated from lung tumors secrete hepatocyte growth factors (HGFs), which in turn activate EMT-related tyrosine–protein kinase Met (c-Met) pathway. Interestingly, they further demonstrated that lung cancer cells with activated c-Met undergo EMT and acquire resistance to tyrosine kinase inhibitors (TKIs) against the epidermal growth factor (EGF) receptor. Giannoni et al. (76) demonstrated that prostate carcinoma-derived interleukin-6 (IL6) activates CAFs, which in turn secrete MMPs, leading prostate cancer (PCa) cells to acquire EMT phenotype and to develop metastases *in vivo* (76). Interestingly, a study proposed by Zhang et al. (77) showed that CAFs could be involved also in organ-specific determination of metastasis. In triple-negative breast cancer, CAFs were described to secrete C-X-C motif chemokine ligand 12 (CXCL12) and insulin-like growth factor-1 (IGF1) factors that promote the expansion of high metastatic clones with high Src activity, a known predictor of bone metastasis (77).

Another key cellular component of TME extensively involved in EMT and metastasis is represented by tumor-associated macrophages (TAMs). TAMs are macrophages that are recruited at the tumor site by tumor-derived chemokines and acquired a tumor-promoting effect (78). In this context, there is evidence that cancer cells can secrete colony-stimulating factor 1 and interleukins in the TME. These molecules can in turn stimulate the production by TAMs of growth factors and MMPs, affecting cancer cell invasion and metastases (78, 79). Using intravital *in vivo* imaging, Condeelis and Segall (80) demonstrated that cancer cells undergoing EMT migrate under the partial guide of the inflammatory microenvironment provided by TAMs. As well as for CAFs, there is evidence that TAMs could promote a specific anatomical site for metastasis development at the premetastatic niche (81).

### Secreted Metabolites

The secretion of active metabolites is an important component of TME communication by supporting tumor metabolism and stimulating tumor growth and metastasis formation (82). The metabolic waste produced by cells in the TME can essentially work as a source of energy or for the synthesis of new molecules. For example, in the condition of low oxygen supply, lactate metabolites generated by anaerobic glycolysis within cancer cells can be posteriorly secreted *via* monocarboxylate transporter 4 (MCT4) that is upregulated under hypoxia (83). Concurrently, adjacent cells can uptake the lactate, *via* MCT1, that will be used to feed the TCA to support energetically the mitochondrial metabolism, showing how lactate can regulate the oxidative metabolism of cancer cells (83). This phenomenon has also been shown to mediate CAFs–cancer cells interactions. Coculture with PCa cells resulted in reprogramming of CAFs toward a Warburg phenotype by upregulation of GLUT1 and MCT4 genes, resulting in the increase of glycolytic activity and secretion of lactate. The lactate is posteriorly metabolized by the PCa cells leading to their growth (84). In addition, lactate has been reported to work as a signaling molecule by suppressing inflammatory responses on immune cells (84).

Similarly, the interaction between cancer cells and CAFs can be mediated by paracrine secretion of amino acids. CAFs, stimulated by ovarian cancer cells, increase glutamine synthesis, that after secretion is used by the same cancer cells, leading to tumor growth (85). Such mutual cross talk was also described with alanine in the interaction between CAFs and pancreatic cancer cells (86). Alanine secreted by CAFs is used by cancer cells for macromolecular biosynthesis, reducing the dependence on glucose and nutrients derived from serum that are usually limited in the TME.

The fatty acids are another class of metabolites that can be released and contribute to tumor propagation. Ovarian and breast cancer cells were shown to be capable to mobilize fatty acids from adipocytes to stimulate tumor proliferation and migration (87, 88). The use of fatty acid as an energy source instead of other molecules (glucose or amino acids) can be dependent on the tumor environment like the presence of adipose tissue or availability of other energy supplies. Moreover, a study correlating obesity and EMT in hepatocellular carcinoma (HCC) revealed that fatty acid uptake *via* fatty acid translocase (CD36) activates Wnt and TGFβ signaling pathways and intensifies HCC progression (89).

### Extracellular Matrix

TME is often seen as a collection of stromal cells that participate in the molecular and phenotypic evolution of cancer. However, it is often overlooked that the most abundant component of TME is represented by the ECM. ECM is a set of structural proteins (such as collagen, laminin, fibronectin, glycans, proteoglycans, and hyaluronic acid) and non-structural secreted enzymes (such as growth factor, hormones, and the family of remodeling proteases). Initially considered as an amorphous scaffold upon which cells are organized, in the past years, ECM has been proposed as a crucial player in tumor progression and diffusion. The involvement of ECM in EMT was anticipated by the seminal paper of Greenburg and Hay (90). They suspended adult and embryonic anterior lens epithelial cells within a mesh of collagen gel. From the morphological point of view, both adult and embryonic epithelial cells developed pseudopodia and filopodia, acquiring a phenotype similar to that of mesenchymal cells *in vivo* (90). From the functional point of view, cells showed an unprecedented behavior for a differentiated epithelium; they became elongated and migrated as single cells within the gel. The author, therefore, concluded that cell interaction with the three-dimensional collagen network is sufficient to promote dissociation and migration and can abolish the original epithelium polarity (90). In addition, they also raised a question: what might be the physiological instructions which regulate the epithelial polarity *in vivo*? They hypothesized that since epithelial surfaces are usually protected by the direct contact with collagens, the disruption of this constraint in pathological conditions could be the accessory driver to EMT. Three decades after this work, Zhang et al. (91) demonstrated that increased stromal deposition of collagen was able to favor the metastatic process in breast cancer. In detail, they observed that the activation of collagen-1 receptor discoidin domain receptor 2 (DDR2) stimulates extracellular signal-regulated kinase (ERK) 2 in a Src-dependent manner, which directly phosphorylates Snai1. Phosphorylated Snai1 accumulates in the nucleus, reduces ubiquitination, and increases the protein half-life. They functionally observed that Snai1 activation induced by DDR2 increased *in vitro* migration and invasion and *in vivo* metastatization of breast cancer cell lines. Finally, they demonstrated that breast cancer cell lines with an invasive phenotype frequently and overexpressing DDR2 associated with nuclear-activated Snai1 and absence of E-cadherin (91). Park et al. (92) highlighted an interplay network between a structural and non-structural component of ECM involved in EMT. In their work, they observed that fibronectin (FN), which is essentially absent in healthy breast tissue but increased in breast cancer, is able to promote EMT *in vitro*. In fact, by exposing MCF-10 cell line to an exogenous combination of FN and TGFβ, they observed an overexpression of EMT markers such as N-cadherin, MMP2, Snai1, phospho-Smad2, vimentin, and α-smooth muscle actin (α-SMA), as well as the acquisition of a cell migratory behavior (92). Also, in this context, the upregulation of EMT genes induced by FN follows the Src kinase/mitogen-activated protein (ERK/MAP) kinase signaling pathways (92).

Hyaluronan (HA) is another component of the ECM that in the TME provides a cancer cell–ECM anchoring site and is associated with a poor prognosis in advanced cancer patients (93). HA exerts its role through the interaction with its membrane receptor CD44, which is frequently overexpressed in different human malignancies. El-Haibi et al. (94) demonstrated that, in the breast cancer cell line, extracellular HA after its binding to CD44 induces CD44 translocation to the nucleus and induction of LOX transcription activation. LOX, in turn, stimulates the transcription of Twist, a well-known marker of EMT. Interestingly, they also demonstrated that *de novo* production of LOX by mesenchymal stromal cells (MSCs) associated to tumor is able to transduce the same signal along the CD44–LOX–Twist axis (94).

In another context, Bourguignon et al. (95) identified a population of CD44^high^-tumor cells exhibiting stem cell-like properties and mesenchymal phenotype able to bind HA through their receptor CD44. This interaction was instrumental in promoting a spheroid formation, as well as cell growth/self-renewal properties in CD44^+^ tumor cells. These findings strongly indicated that HA–CD44 interaction in the ECM of highly metastatic cancer cells is correlated with the transcription of stem cell markers which are important contributors to the head and neck squamous cell carcinoma progression (95).

Between the non-structural components of ECM, periostin is one of the most known factors contributing to EMT. Bao et al. (96) demonstrated that periostin is strongly upregulated in the majority of colorectal cancers, with the highest index of expression in metastatic tumors. They observed that exogenous overexpression of periostin in colorectal cancer cell lines promotes liver metastasis growth *in vivo*, reduces stress-induced apoptosis, and enhances neo-vascularization. Finally, by exploring the molecular mechanism by which periostin promotes EMT, they observed that periostin activates the Akt/PKB kinase through the interaction with αvβ3 and the αvβ5 integrins (96). In this work, they concluded that “The life and death decision at the cellular level is controlled by the proper cell–matrix interactions” (96). Kim et al. (97) added some captivating alternative results on the behavior of periostin in different tumors. Periostin resulted down-expressed in PCa but overexpressed in bladder cancer. In bladder cancer, periostin antagonizes EMT by downregulating Twist. On the contrary, in PCa, periostin upregulates Snai1 favoring EMT (97). During tumor progression, ECM is submitted to continuous remodeling by different types of stromal cells. In this context, the cross-linking of collagen mediated by LOX plays a crucial role in the EMT process. Paszek et al. (98) noted a possible link between the increased stiffness observed in tumor and the mechanotransduction ability of cells, mainly through integrins. In their work, they demonstrated that the substrate's stiffness in which cells are cultivated affects epithelial morphogenesis by an integrin-mediated ERK activation and increases contractility and focal adhesions mediated by the activation of ROCK (98). Levental et al. (99) found that breast cancer progression goes in parallel with collagen cross-linking and ECM stiffening. They demonstrated that a stiffer ECM promotes focal adhesions, enhances phosphoinositide (PI)3 kinase activity, and induces EMT (99). Once again, these data confirm that the non-cellular part of TME plays a fundamental role in the extrinsic regulation of EMT.

The mechanical signals including ECM rigidity, shape and porosity, cell matrix adhesion, cell geometry, and cytoskeletal tension can regulate EMT by affecting the YAP/TAZ signaling. Mechanical tension has been demonstrated to stimulate a β-catenin-independent YAP triggering, involved in cell cycle progression through G1 into S phase (100). In this context, it has been recently demonstrated that changes in the TME architecture can play a crucial role in the oncogenic mechanotransductional regulation of YAP/TAZ signaling through Rac1 activation (101).

Although interesting results are yet present, many efforts still have to be made to understand how to use these molecules as a diagnostic, prognostic, or predictive biomarker. Finally, since the TME component of cancer is less susceptible to the evolutionary pressure of cancer cells, which leads to therapy resistance, these components could also be used as therapeutic targets to inhibit EMT.

### Extracellular Vesicles

Since the seminal formulation of the “seed-and-soil” hypothesis by Paget (102), significant advances in research have made it possible to demonstrate the molecular mechanisms of cancerous metastasis and the complex interplay between cancer cell and the host microenvironment. Studies on extracellular vesicles (EVs), first identified as a garbage disposal (103, 104), demonstrated their ability to transferring biological information from donor to receiving cells in both physiological and pathological processes (105). The EV cargo molecules cover all the spectrums of bioactive molecules such as proteins, lipids, oncogenic virus-derived molecules, microRNAs (miRNAs), mRNAs, and DNA fragments (106), allowing them to exert an autocrine, paracrine (when they diffuse to neighboring cells), and endocrine (when they are carried *via* systemic transport) signaling transduction on organ-specific locations or recipient cells. A body of evidence suggests that tumor-derived EVs or EVs secreted by CAFs in the TME play a fundament role in triggering EMT, tumor invasion, and metastasis (107, 108). The direct role and mechanism of action of EVs in tumor are described along this review and resumed in Table 1. Furthermore, referring to the “seed-and-soil” hypothesis, the major breakthrough in the tumor-derived EV research was the preliminary observation of their possible role as “fertilizer” in the organ-specific determination of pre-metastatic niches (81, 128, 129). In this context, the ability of tumor-derived EVs to use systemic circulation induces the organotropism of metastatic tumors and promotes the pre-metastatic niche formation by showing “avidity” for specific recipient cells (109, 110).

**Table 1:** Summary of the studies highlighting the active role of EVs as tool and effector in TME remodeling, EMT, and pre-metastatic niche preparation.

**References**	**Sources**	**Activated pathways**	**Biological function**
**Tumor-derived EVs**			
Hoshino et al. (109)	TDEs from human breast and pancreatic cancer cell lines	TDE-derived integrins activate Src phosphorylation and pro-inflammatory S100 gene expression in resident fibroblast and endothelial cells	Stimulation of the preparation of the pre-metastatic niche
Costa-Silva et al. (110)	Exosomes from pancreatic ductal adenocarcinomas (PDACs)	PDAC-derived exosomes induce TGFβ secretion by Kupffer cells and upregulation of FN production by hepatic stellate cells	Induction of liver pre-metastatic niche formation in naive mice and increase in liver metastatic burden
Franzen et al. (111)	Exosomes from T24 or UMUC3 invasive bladder	TDEs induce overexpression of α-SMA, S100A4, and Snai1 in of Non-invasive urothelial cells	Increase of the migration and invasion ability
Rahman et al. (112)	TDEs from serum of lung cancer patients and from lung cancer metastatic cells (PC14HM cells)	Serum-derived TDEs induce the upregulation of N-cadherin and the reduction of E-cadherin and ZO-1 expression in exosome treated HBECs	Induction of migration, invasion, and proliferation
Chen et al. (113)	Exosomes from highly metastatic hepatocarcinoma cells (MHCC97H)	TDEs induce the activation of EMT *via* MAPK/ERK signaling pathway in low metastatic HCC cells	Stimulation of the migration, invasiveness, and chemotactic ability
Zhou et al. (114)	Exosomes from metastatic breast cancer cells	Exosomes transfer miR-105 to endothelial monolayer cells	Elimination of tight junctions and induction of vascular permeability promoting metastasis in distant organs
Hakulinen et al. (115)	EVs released from human melanoma and fibrosarcoma cells	Exosomes containing MT1-MMP activate pro-MMP-2 and degrade type 1 collagen and gelatin	Direct effects on ECM composition regulating tissue homeostasis and cell invasion
Graves et al. (116)	Malignant ovarian tumor-derived EVs	EVs carry specific kallikreins and MMP inducers	Contribution to matrix degradation that facilitates tumor cell invasion and metastasis
**EMT–modified EVs**			
Garnier et al. (117); van Hinsbergh et al. (118); Garnier et al. (119)	Exosomes from EMT-derived epithelial cancer cells (A431 and DLD-1)	Modification of EV proteome with the upegulation of different proteins such as TF, integrin α2, and CD9	Induction of a switch from classical endothelial anticoagulant properties
Tauro et al. (120)	Exosomes from Ras-transformed MDCK cells	EMT-derived exosomes are enriched with several proteases, integrins, transcriptional regulators (e.g., the master transcriptional regulator YBX1), and core splicing complex components	Induction of invasive and mesenchymal phenotype in recipient epithelial cells
Karaosmanoglu et al. (121)	Exosomes derived from Slug overexpressing HCC-derived HepG2 and Huh7 cells	Exosomes express elevated levels of posttranslationally modified FN1, COL2A1, and native FGG	Induction chemoresistance and EMT in HCC
Hardin et al. (122)	Exosomes derived from TGFβ-treated and CSC subpopulation of papillary thyroid carcinoma (PTC) cell lines	Exosomes induce the upregulation of the lncRNA MALAT1 and the transcription factors SLUG and SOX2 in the normal thyroid cells	Involvement in TGFβ pathway and cell motility; reprogramming of the pre-metastatic niche
**Cancer stem cell–derived EVs**			
Grange et al. (123)	Renal CD105^+^ CSC-derived EVs	CSC-derived EVs contain proangiogenic mRNAs and microRNAs potentially implicated in metastasis formation. CSC-derived EVs induce the expression of VEGFR1, VEGF, MMP9, and MMP2 in lungs of SCID mice.	Activation of angiogenesis and stimulation of lung metastasis formation
Lindoso et al. (124)	Renal CD105^+^ CSC-derived EVs	CSC-derived EVs promote an increased expression of genes associated with cell migration (CXCR4, CXCR7), matrix remodeling (COL4A3), angiogenesis, and tumor growth (IL-8, osteopontin and myeloperoxidase) in MSCs	Enhancement of migration in EV-stimulated MSCs. EV-stimulated MSCs became pro-angiogenic
**TME–derived EVs**			
Luga et al. (125)	Exosomes from CD81-positive CAF subpopulation	CD81-positive exosomes promote breast cancer cell autocrine release of Wnt11 and activation of Wnt-planar cell polarity (PCP) signaling pathway	Induction of cancer protrusive activity and motility
Donnarumma et al. (126)	Breast CAF-derived exosomes	CAF-derived exosomes induce the increase in the expression of stem cell and EMT markers in breast cancer cell lines through the transfer of miR-21, –378e, and –143	Stimulation of the capacity to form spheroids and the anchorage-independent cell growth
Li et al. (127)	Ovarian CAF-derived exosomes enriched of TGFβ1	SKOV-3 and CAOV-3 cell lines treated with CAF-derived exosomes activate the SMADs signaling pathway by inducing phosphorylation of SMAD2/3 complex	Enhancement of invasion capability and EMT promotion

*TDEs, tumor-derived exosomes; FN, fibronectin; α-SMA, α-smooth muscle actin; Snai1, Snail family transcriptional repressor 1; HBECs, human bronchial epithelial cells; HCC, hepatocarcinoma cells; MT1-MMP, membrane-type 1–matrix metalloproteinase; TF, tissue factor; MDCK, Madin–Darby canine kidney; COL2A1, collagen type II alpha 1; FGG, fibrinogen gamma chain; CSCs, cancer stem cells; MSCs, mesenchymal stem cells; CAFs, cancer-associated fibroblasts; EMT, epithelial to mesenchymal transition; TGFβ, transforming growth factor-beta 1; VEGF, vascular endothelial growth factor; VEGFR, vascular endothelial growth factor receptor; MMPs, matrix metalloproteinases; CRCRs, C-X-C chemokine receptors; COL4A3, collagen type IV alpha 3 chain; Wnt11, Wnt family member 11*.

In recent years, tumor-derived exosomes (TDEs), a particular subclass of EVs is largely involved in EMT plasticity (107). Exosomes are 30–100-nm-diameter vesicles secreted into the extracellular space through fusion with the cell membrane (106). Franzen et al. (111) isolated exosomes from patient urine and bladder barbotage and from invasive bladder cancer cell-conditioned media. Noninvasive urothelial cell treated with isolated TDEs showed overexpression of S100 calcium-binding protein A4 (S100A4), Snai1, and α-SMA compared with control cells. Moreover, the treatment of non-invasive urothelial cell with bladder cancer TDEs functionally increased their migration and invasion ability (111). Similarly, Rahman et al. (112) isolated TDEs from serum of lung cancer patients and both from cancer non-metastatic and metastatic cells. They demonstrated that TDE derived from highly metastatic lung cancer cells and serum from late-stage lung cancer patients induced EMT through vimentin overexpression in human bronchial epithelial cells. Furtermore, they functionally showed that both sources of TDEs induce migration, invasion, and proliferation in the same cell type (112).

Interestingly, some work highlighted that also stromal cells secrete exosomes that could promote EMT in cancer cells. Luga et al. (125). reported that TDEs released by the specific CD81-positive CAF subpopulation induced breast cancer motility by the autocrine release of Wnt11 from the breast cancer cell line. Donnarumma et al. (126) demonstrated that CAF-derived exosomes are enriched in the content of miRNA-21, –378e, and –143 compared to normal fibroblasts. Breast cancer cell lines exposed to CAF-derived exosomes significantly increased their capacity to form spheroids with the expression of stem cell and EMT markers compared to normal fibroblast exosome exposure (126). Finally, Li et al. (127) demonstrated that ovarian CAF-derived exosomes contained a higher level of TGFβ1 compared to normal omentum fibroblasts. SKOV-3 and CAOV-3 cell lines treated with CAF-derived exosomes showed an enhanced invasion capability and the EMT promotion through the activation of a SMAD signaling pathway (127).

## Epithelial to Mesenchymal Transition as a Modulator of the Tumor Microenvironment

**Figure 3:**
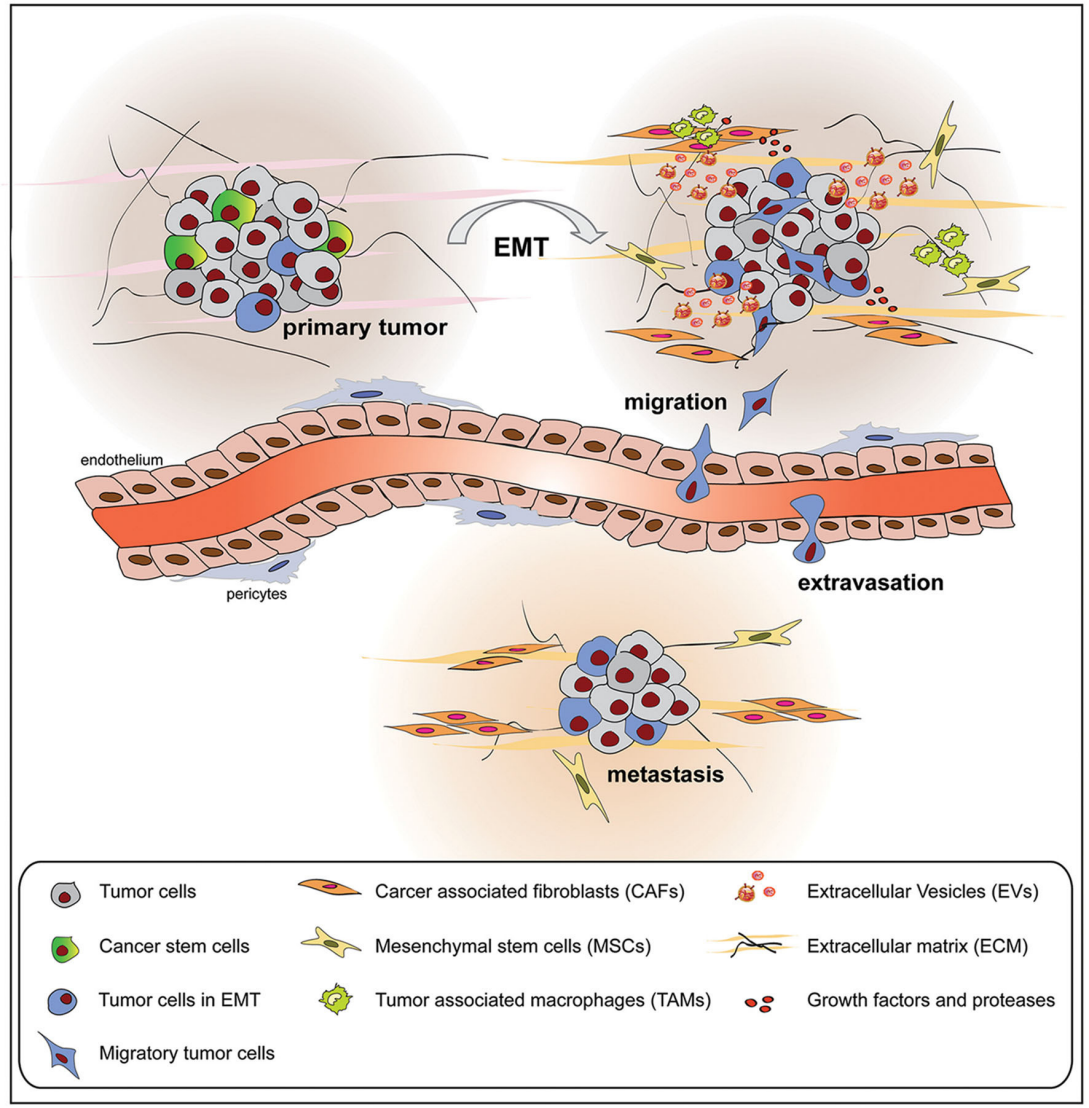
Tumor cells undergoing epithelial to mesenchymal transition (EMT) interact with the surrounding tumor microenvironment (TME), composed of cellular and non-cellular components, modifying its composition and metabolic properties. The mutual influence between tumor cells and TME promotes tumor growth, extravasation, and metastasis formation.

During the EMT, tumor cells contribute to several interactions with the surrounding TME, composed of cell components and ECM as well as cell secretome elements (130). As a result, the TME is also modulated by the EMT process toward tumor progression and metastasis (Figure 3). In this section, we focused our attention on the non-cell-derived components, such as ECM as well as EVs, on their modifications and how they can act as suitable tools for anticancer therapy.

### Epithelial to Mesenchymal Transition Promotes Extracellular Matrix Modification

In cancer, as described above, ECM remodeling plays an important role by affecting different processes such as tumor growth (99) and metastasis induction (131). During the activation of EMT, tumor cells are submitted to mechanical perturbations mediated by the activation of specific oncogenes (132). This molecular pattern stimulated tumor cells to respond alternatively to the microenvironment stimuli, enhancing their actin cytoskeleton arrangement and affecting ECM composition (132). To invade, tumor cells undergoing EMT should remodel the ECM by enhancing the secretion of proteases as well as large scaffolding proteins by affecting their migratory potential (8). Scaffolding proteins embrace molecules such as FN1, plasminogen activator inhibitor 1 (PAI1), collagens, and periostin and composed the ECM network, providing molecular signals and tensional forces that are required to the cell to migrate. The modification in ECM conformation determines changes in physical parameters, such as stiffness and network dimension, that can reveal the clinical diagnosis of cancer progression (133–135).

Recently, Peixoto et al. (136) identified a specific epigenetic signature in genes associated with ECM remodeling in three different tumor models of EMT (lung, breast, and renal tumors). Interestingly, the epigenetic signature identified in seven genes was identical independently from the cancer cell model and the EMT inducer (136). The authors identified *ADAM19* and its epigenetic regulation as a robust new biomarker of EMT *in vitro* and *in vivo* (136). With another experimental approach using 3D biomimetic scaffolds, Liverani et al. (137) demonstrated that metastatic breast tumors have a higher capability to alter the extracellular collagen structures, affecting the mechanobiology of cell-ECM in metastatic tumors. Moreover, the modification in collagen hydroxylation state was associated with pyruvate metabolism in metastatic breast cancer cell lines (138). The authors demonstrated that pyruvate functions as an essential nutrient in the pre-metastatic niche by inducing ECM modification and metastasis formation (138).

The extracellular matrix-remodeling enzyme LOX, when secreted, controls the tissue flexible force through the catalysis of elastin and collagen cross-linking (48). LOX-mediated collagen cross-linking was demonstrated to affect tissue fibrosis and matrix stiffness, enhancing cell metastatic colonization and growth *in vivo* in breast (99), colorectal (139), and lung cancers (140). Interestingly, the expression of LOX was found significantly correlated with MMP2/MMP9 expression in metastatic non-small-cell lung carcinoma (NSCLC) (141).

Alterations in ECM composition have been related to metastatic niche formation in numerous tumors (142). The proteins identified such as cell adhesion molecules (CAMs), MMPs and collagens, specific CXC motif-chemokines (CXCLs), and citrullinated proteins/PAD have a different biological function, and their alterations depend on the originating primary tumor (142). Different reports identified in colorectal liver metastatic patients included elevated levels of type I collagen in urine (143, 144) and blood (145). These results not only demonstrate that the active turnover of specific collagens in the liver is propaedeutic to the preparation of the soil for tumor seeding in the metastatic niche but also suggest the use of these molecules as metastasis biomarkers (146). In the bone, metastatic cancer cells in the first stage after extravasation are quiescent and mimic osteoblasts to escape immune surveillance (147). During this phase, cancer cells recruit the surrounding bone-derived osteoclasts and fibroblasts and indirectly modify collagens and fibronectin (FN) deposition, thus leading to ECM disorganization (148).

Endoplasmic reticulum (ER) stress signaling has been strongly connected to cancer cell migration and invasion through adaptive stress responses that include the unfolded protein response (UPR) (149). UPR activation induced morphological changes in cancer cells that modulated classical EMT markers such as vimentin and E-cadherin (150, 151). Furthermore, protein kinase RNA-like ER kinase (PERK)–eukaryotic initiation factor 2 (eIF2α)–activating transcription factor 4 (ATF4) axis, which is involved in the ER homeostasis, has been directly associated to EMT-dependent invasion and metastatic processes (152) by supporting the synthesis of ECM components, i.e., collagens and FN, and enzymes, i.e., cathepsin and MMPs, involved in ECM remodeling (152, 153).

### Epithelial to Mesenchymal Transition Shifts Extracellular Vesicles Into a Pro-metastatic Phenotype

EMT process is strongly associated with the change in the cellular secretome including EVs. EVs as carriers of molecular information have been demonstrated to influence the EMT process. In a complementary way, tumor cells undergoing EMT can generate EVs able to modify the surrounding TME (154). In particular, EMT induction in epithelial cancer cells (A431 and DLD-1) has been demonstrated to induce quantitative changes in the vesiculation pattern of EMT-induced A431 cells, with the stronger release of procoagulant exosomes enriched of tissue factor (TF) (117). Such EMT-derived EVs can interact with endothelial cells causing a switch from their classical anticoagulant properties (118). The same group demonstrated that the induction of EMT by E-cadherin blockade and EGFR stimulation enrich in a subpopulation of the CD44^high^/CD24^low^ tumor cells (119). This modification was reflected also in the EV proteome through the detection of 30 unique proteins and the enrichment of pathways related to cellular growth and migration (119). Furthermore, Tauro et al. (120) demonstrated a proteome modification in exosomes derived from mesenchymal Madin–Darby canine kidney cells reprogrammed with H-Ras (21D1 cells), with enrichment of classical mesenchymal markers both in the cells and their released exosomes. Several MMPs and integrins implicated in regulating metastatic progression were also compartmentalized in 21D1 exosomes (120). Additionally, the authors observed an enhancement of transcription factors that function as EMT inducers in 21D1 exosomes, suggesting a spread of the invasive and mesenchymal phenotype through EVs in cancer (120). In another work, exosomes derived from Slug-overexpressing HCC cells were shown to express elevated levels of posttranslationally modified FN1, collagen type II alpha 1 (COL2A1), and native fibrinogen gamma chain (FGG) (121). These molecules have been proposed as non-invasive biomarkers for chemoresistance and EMT in HCC. In a thyroid cancer model, the treatment of papillary thyroid carcinoma cell line (TPC1) with TGFβ was able to induce the release of a higher number of exosomes in respect to the untreated cells (122). Moreover, exosomes derived from TGFβ-treated cells significantly upregulated the long non-coding RNA (lncRNA) MALAT1 and the transcription factors SLUG and SOX2 in naive treated cells, involved in TGFβ pathway and cell motility (122). In another study, Chen et al. (113) demonstrated that through the activation of EMT *via* mitogen-activated protein kinase (MAPK)/ ERK signaling pathway, highly metastatic hepatocarcinoma cells (MHCC97H) are able to similarly stimulate the migration and invasiveness ability of low metastatic hepatocarcinoma cell line.

Recently, different reports have shown that the formation of a pre-metastatic niche depends on tumor-derived EVs and their capacity to modulate adjacent and distant TME (155, 156). Grange et al. (123) demonstrated that renal CD105^+^ CSCs can release EVs able to activate angiogenesis and enhance lung metastasis, accounting for the preparation of a pre-metastatic niche in the lung. The same EVs were able to educate MSCs to a pro-tumorigenic phenotype by stimulated pro-angiogenetic and pro-invasive actions (124). Interestingly, the long intergenic non-coding RNA regulator of reprogramming (linc-ROR) was transferred from thyroid CSCs to normal thyroid cells *via* exosomes, mediating the induction of EMT and the reprogramming of the metastatic niche (122).

EVs can affect all the components of the TME. In breast cancer, exosomes from metastatic breast cancer cells transferred miR-105 to endothelial monolayer cells. Exosome treatment efficiently destroys tight junctions and induced vascular permeability promoting metastasis in distant organs (114). Shu et al. (157) discovered the ability of human melanoma-derived exosomes to reprogram adult dermal fibroblast metabolism by increasing glycolysis, thus contributing to the favorable conditions for the pre-metastatic niche. Lastly, Costa–Silva et al. (110) demonstrated that pancreatic ductal adenocarcinoma-derived exosomes can function at the level of hepatic stellate cells which in turn upregulate the TGFβ and FN production and enhance the recruitment of bone marrow-derived macrophages. The synergistic activity of a fibrotic microenvironment combined with pro-tumor recruitment of bone marrow-derived macrophages suggests an active role of pancreatic ductal adenocarcinoma-derived exosomes in directing pancreatic cancer cells toward metastatic liver priming (110).

Huleihel et al. (158) recently showed that biologic scaffolds can contain functional EVs bound by matrix components supporting that EVs can be classified as structural and active components of the ECM that participate in matrix organization as well as contributing to the physical properties of ECM. Hakulinen et al. (115) demonstrated the capability of EVs released from human melanoma and fibrosarcoma cells to affect the ECM composition through the enzymatically active membrane-type 1–matrix metalloproteinase (MT1-MMP). In addition, malignant tumor-derived EVs carry also specific kallikreins and MMP inducers that also contribute to matrix degradation, which may facilitate tumor cell invasion and metastasis (116).

## New Tools for the Treatment of Tumor Microenvironment Components

### Targeting the Tumor Extracellular Matrix

In recent years, studies applied to the biomarkers field for identifying diseases and new therapeutic options have grown exponentially (159). EMT has been demonstrated to play a relevant role in numerous phases of tumor development such as induction of CSC phenotype, resistance to apoptosis, migration, and metastasis formation, being a promising therapeutic target in cancer (160). However, the possibility to directly affect the key molecules involved in EMT is challenging.

An alternative approach followed in different preclinical studies is to directly target the EMT-inducing TME to interfere in cancer metastatic and invasive profiles (160). Both the formation of pre-metastatic niche and the following tumor migration to distant sites have been strongly implied in the involvement of ECM. For this reason, tumor ECM has been proposed as a possible target for anticancer therapy. The use of methylumbelliferone, an HA synthesis inhibitor supplemented with zoledronic acid, a conventional therapeutic agent for bone metastases, was more effective than the single therapies in suppressing proliferation, migration, and invasion of murine lung carcinoma cell lines (161). Moreover, the combination therapy showed a stronger effect in the reduction of metastatic bone lesions *in vivo* (161). The use of a neutralizing antibody directed against the ECM component periostin (PN1-Ab) produced by mouse breast cancer cells significantly inhibited tumor cell proliferation and invasion. Moreover, the administration of PN1-Ab prolonged cell survival through inhibition of the lung metastasis formation *in vivo* (162). In the same pathological context, targeting the hypoxia-dependent ECM enzyme LOX suppresses migration and invasion of MDA-MB-231 cells in a FAK-dependent inhibition mechanism (163). In addition, the use of epigenetic drugs, targeting EMT-induced epigenetic modification in the ECM component *ADAM19* (136), has been proposed as an anticancer therapy in combination with conventional drugs in metastatic NSCLCs. Affecting ECM components with pharmacological approaches based on single molecules already gave disappointing results or limited benefits as reported by Paolillo and Schinelli (142). For this reason, almost all the different approaches hypothesized to neutralize metastasis formation are combination treatments, as already described for other pathologies, that are affecting multiple signaling pathways and TME components involved in the metastatic process (160).

Another approach proposed is the use of ECM molecules for drug delivery purposes, taking advantage of unique modifications induced by EMT in the TME, to achieve a potent and selective delivery system. For example, engineered HA-based conjugates have emerged as a promising strategy to efficiently target tumors with drugs exerting poor solubility and strong side effects (164). Lee et al. (165) demonstrated that HA-conjugated paclitaxel (PTX) strongly inhibited proliferation and increased apoptosis of ovarian cancer cells in xenograft nude mice. Moreover, HA-PTX increased the survival rate of mice, significantly reduced the density of microvessels in tumor tissues, and eliminated ascites formation in transplanted ovarian cancer cell lines in the same model (166). HA-mediated targeting of intracellular nucleic acids and other drugs has great potential for clinical application (166). Park et al. (167) demonstrated that intratumoral injection of vascular endothelial growth factor (VEGF) siRNA conjugated with modified HS molecules dramatically reduced tumor growth and directly regulated tumor vasculature and VEGF expression in melanoma tumors.

### Targeting the Tumor Extracellular Vesicles

**Figure 4:**
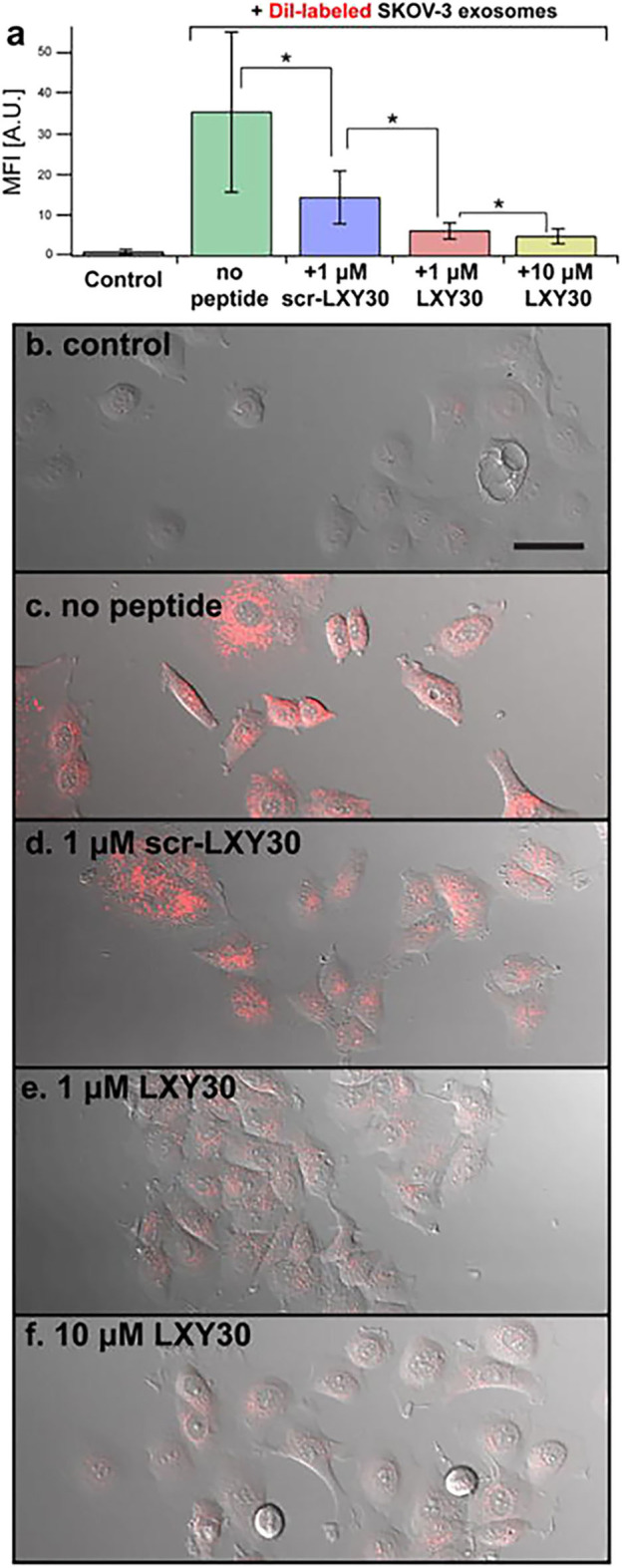
Example of a therapeutic approach based on the targeting of tumor extracellular vesicles (EVs). Inhibition of the EV uptake by SKOV-3 cells treated with increased concentrations of LXY30 peptide **(A)** by measuring fluorescence intensity in target cells *p < 0.005. **(B–F)** Representative laser scanning microscope images of SKOV-3 cells treated with unlabeled **(B)** or labeled DiL-EV in combination with different concentrations **(C–F)** of LXY30 peptide. Contribution from Carney et al. (168). Copyright Wiley-VCH Verlag GmbH & Co. KGaA. Reproduced with permission.

The capabilities of EVs to “educate” distant cells and prepare the so-called “soil” for metastasis formation classified EVs as a therapeutic target for cancer treatment. A block in EV uptake can represent a strategy to affect EV capability to modify TME and prepare the pre-metastatic niche. Using this approach, Carney et al. (168) demonstrated the capacity of LXY30 peptide to selectively bind α3β1 integrin overexpressed on tumor cell-derived exosomes. LXY30-modified Dil-EVs from ovarian cancer cells reduced cell uptake up to ~80% in respect to their naive counterpart (168) (Figure 4). The selectivity of LXY30 to tumor EVs and not to non-tumor EVs strongly improves its application in therapeutic and diagnostic nanomedicine (168).

The transfer of genetic information (ncRNAs and mRNAs) through cancer and non-cancer EVs is a potential mechanism of intercellular communication in TME. Therefore, modulating or changing the genetic content of EVs might be a strategy to prevent cancer propagation. In this direction, EVs have also been proposed as a delivery carrier for antitumor molecules. This is in line with previous reports that demonstrated that naive EVs from different sources of MSCs were able to exert an antitumor activity in different cancer cell models (169–171). By applying modification in the EV molecular content, Ota et al. (172) observed that miR-30e-enriched EVs can reduce proliferation and invasion of cholangiocarcinoma cell (CCA) through direct regulation of EMT (172). Moreover, EVs carrying miR-379 generated from engineered MSCs impaired metastatic breast cancer growth *in vivo*, acting partially through the regulation of COX-2 (173).

### Extracellular Vesicles as Fingerprint of Tumor Metabolic State

Recent studies have proposed the use of tumor-derived EVs as tools for cancer detection and monitoring (174). EVs are released from the primary tumor and metastasis, and they can be directly detected in biofluids, providing real-time non-invasive tumor tracking. Using the next-generation sequencing (NGS) technology, Möhrmann et al. (175) demonstrated that the detection of common mutations (BRAF, RAS, and EGFR) in plasma exosomal nucleic acids (exoNAs) showed a stronger sensitivity compared with other clinical testing plasma cell-free DNA (cfDNA). The same approach was applied by using another biological matrix as a source of EVs, the urine. Clos–Garcia et al. (176) were able to detect selective metabolites in urine EVs of PCa patients. They also demonstrated that the most elevated metabolites detected in PCa urine EVs were correlated to steroid biosynthesis and associated with increased perineural invasion (176). Interestingly, EVs isolated from tumor proximal body fluids can offer a real representation of the metabolic alterations occurring at the tumor site. In endometrial cancer (EC), Martinez–Garcia et al. (177) identified a uterine fluid aspirate-based EV protein signature to diagnose EC and classify tumors subtypes. In another study, Sequeiros et al. (178) identified a set of proteins in urinary EVs able to discriminate high- and low-grade PCa, reflecting the histological changes in tumor specimens. This study underlines the potential to produce a new diagnostic assay for EV-based liquid biopsies using proteomic approaches for the diagnosis and prognosis of PCa (178).

MiRNAs isolated from tumor-derived EVs are more stable and therefore considered to be more reliable biomarkers (179). In addition, EVs containing lncRNAs are also other attractive options as diagnostic/prognostic factors for cancer progression (180). Baumgart et al. (181) identified a miRNA panel in tumor tissues as well as in urinary EVs able to discriminate muscle-invasive bladder cancer (MIBC) patients from healthy individuals. Similarly, Roman–Canal et al. (182) characterized a cohort of miRNAs in EVs derived from ascitic liquid and peritoneal lavages of colorectal cancer (CRC) patient as promising biomarkers for CRC diagnosis. Berrondo et al. (183) identified HOTAIR and other tumor-associated lncRNAs in the urinary exosomes derived from urothelial bladder cancer (UBC). Loss of HOTAIR was able to reduce the expression of EMT genes as well as *in vitro* invasive properties in UBC cell lines, suggesting a cardinal role for exosome-derived HOTAIR in tumor initiation and progression (183).

Recent studies have demonstrated the enrichment of specific surface molecules in EVs derived from a variety of tumor cells, accounting for their use as disease biomarkers. Sharma et al. (184) demonstrated melanoma tumor-derived exosomes (MTEX) contain on their surface a set of melanoma-associated antigens (MAAs) that were not detectable in exosomes produced by normal cells. By applying immune-based capture technique for the CSPG4 epitope, the authors identified a specific protein profile of MTEX that was qualitatively and quantitatively distinct from normal cell-derived exosomes in plasma of patients with melanoma. In a similar way, Bai et al. (185) recently developed an immuno-based microfluidic chip using a quantum dot multiplex detection system to characterize lung cancer exosomes for the expression of different cancer-related surface molecules.

Ideally, for cancer diagnostic purposes, the development of tools to identify biomarkers in tumor–EV subpopulations should be designed, considering the following needs: (i) a sensitive detection method that considers the small proportion of tumor-derived EVs in respect to the total non-tumor secretome in the body fluids; (ii) the selectivity in the chosen molecules that can separate tumor from non-tumor EV background; and (iii) the representativity in the tumor metabolic state that can discriminate stages and progression/relapse during the clinical treatment.

## Conclusion

In cancer, EMT appears as a key pathological process characterized by abnormal metabolic reprogramming of cancer cells toward an invasive and pro-metastatic phenotype. The signals responsible for the activation of the EMT can derive directly from an alteration of cancer cell metabolism. However, extrinsic inductors, coming from the cellular and non-cellular TME components, can directly participate in the EMT process modulating cancer cells toward a pro-tumorigenic and pro-invasive phenotype. In turn, tumor cells undergoing EMT modify the surrounding environment that actively helps the tumor to invade and metastasize. The non-cell TME-derived components, participating actively in the tumor spread, have been proposed as possible tools for cancer monitoring and treatment, being on one side biomarkers of disease regression/relapse (i.e., EVs) and on the other side possible direct targets for new-combination anticancer personalized medicine.

## Author Contributions

FC and MA planned the manuscript. ED'A, RL, FS, and FC contributed in writing the manuscript and screened international scientific literature. BB and SP helped in revising the manuscript. FC designed the figure. All authors approved the submitted version.

## Conflict of Interest

The authors declare that the research was conducted in the absence of any commercial or financial relationships that could be construed as a potential conflict of interest.

## Abbreviations

EMT: epithelial to mesenchymal transition
TME: tumor microenvironment
EV: extracellular vesicle
MMP: matrix metalloproteinase
CSC: cancer stem cell
GLUT: glucose transporter
HIF-1α: hypoxia-inducible factor 1α
ZEB: zinc finger E-box-binding homeobox
HK: hexokinase
ALDO: aldolase
GAPDH: glyceraldehyde-3-phosphate dehydrogenase
TCA: tricarboxylic acid cycle
SDH: succinate dehydrogenase
TGFβ: transforming growth factor beta
ACSL: acetyl-CoA synthetase
SCD: stearoyl-CoA desaturase
ACL: ATP citrate lyase
LOX: lysyl oxidase
IDO: indoleamine 2,3-dioxygenase
YAP: YES-associated protein
TAZ: transcriptional coactivator with PDZ-binding motif
SREBP: sterol regulatory element-binding protein
AKT: protein kinase B
CAF: cancer-associated fibroblast
HGF: hepatocyte growth factor
TKI: tyrosine kinase inhibitor
EGF: epidermal growth factor
CXCL: C-X-C motif ligand
IGF: insulin growth factor
TAM: tumor-associated macrophage
MCT: monocarboxylate transporter
HCC: hepatocellular carcinoma
CD: cluster of differentiation
DDR: discoidin domain receptor
α-SMA: alfa-smooth muscle actin
HA: hyaluronic acid
TDE: tumor-derived exosome
ECM: extracellular matrix
FN: fibronectin
PAI: plasminogen activator inhibitor
NSCLC: non-small-cell lung carcinoma
CAM: cell adhesion molecule
UPR: unfolded protein response
PERK: protein kinase RNA-like ER kinase
TF: tissue factor
COL: collagen
PN: periostin
VEGF: vascular endothelial growth factor
MSC: mesenchymal stem cell
exoNA: exosomal nucleic acid
cfDNA: cell-free DNA
miRNA: microRNA
lncRNA: long non-coding RNA
MIBC: muscle-invasive bladder cancer
PCa: prostate cancer
EC: endometrial cancer
MIBC: muscle-invasive bladder cancer
CRC: colorectal cancer
UBC: urothelial bladder cancer
MTEX: melanoma tumor-derived exosome
MAA: melanoma-associated antigen
E-cad: E-cadherin.
